# The relationship between family socioeconomic status and cultural background on the career self-determination of Chinese and Kazakhstani students

**DOI:** 10.3389/fpsyg.2026.1849182

**Published:** 2026-06-22

**Authors:** Xiaogang Jin, Zhan Zhou, Chunling Wang, Xiaoxing Hu, Junxian Li, Xintian Lyu, Aigul Alchimbayeva

**Affiliations:** 1 Faculty of Philosophy and Political Science, Al-Farabi Kazakh National University, Almaty, Kazakhstan; 2 Faculty of Arts, Huanggang Normal University, Huanggang, China

**Keywords:** career self-determination, China-Kazakhstan cross-cultural comparison, cultural values, family socioeconomic status, structural equation model

## Abstract

**Introduction:**

The primary objective of this quantitative cross-cultural study is to precisely investigate how multi-dimensional objective family capital, subjective social status, and cultural values are jointly associated with the career agency of youth. Career self-determination is crucial for youth development, yet the factors associated with it are culturally specific. Grounded in Self-Determination and Social Cognitive Career Theories, this study aims to explore these complex relationships.

**Methods:**

This study examines the interplay of family socioeconomic status (SES) and cultural values regarding the career self-determination of 1,020 students in China and Kazakhstan via a structured survey. Data were analyzed using structural equation modeling (SEM), multi-group comparisons, and relative weight analysis (RWA).

**Results:**

Key findings reveal a significant structural difference in the association of family capital, rooted in differing national contexts (involutionary competition vs. reliance on relational networks). For Chinese youth, economic capital is the primary factor associated with career self-determination, whereas for Kazakhstani youth, social capital is paramount. Furthermore, the study establishes that subjective social class perception acts as a key psychological mediator in the relationship between objective family capital and career self-determination. While traditional values exhibit a direct negative association with self-determination across both cultures, they do not moderate the SES association.

**Discussion:**

These findings enrich career development theories by highlighting their contextual boundaries in non-Western transitional societies and offer vital, culturally-sensitive guidance for youth career counseling policy within the “Belt and Road” initiative.

## 1 Introduction

Career self-determination, defined as the intrinsic motivational capacity and autonomous agency an individual demonstrates when independently making and executing career choices, is not only profoundly related to the immediate career adjustment of adolescents but also significantly associated with their long-term career adaptation and subjective well-being (McAnally and Hagger, 2024; Ryan et al., 2025). From a macro ecological systems perspective, family, and culture are widely regarded as important contextual factors associated with the development of this psychological capacity (Bronfenbrenner, 1979; Markus and Kitayama, 2010). Through the economic support, educational resources, and social networks it can mobilize, the family builds an undeniable material and capital foundation for an individual's career choices (Bourdieu, 1986). Here, family socioeconomic status (SES) is broadly conceptualized as the comprehensive economic, social, and cultural resources accessible to an individual through their family background. Meanwhile, culture, through its values, behavioral norms, and collective expectations rooted in the social fabric, provides career choices with a specific “meaning framework” and boundaries of rationality (Markus and Kitayama, 2010).

Against the grand backdrop of the “Belt and Road” Initiative, China and Kazakhstan, as geographically adjacent and increasingly interactive important nations, exhibit commonalities and particularities in the career development of their youth that are worthy of in-depth investigation. Recent statistical evidence highlights the urgency of this issue: amid rapid macroeconomic restructuring, substantial proportions of university graduates in both nations report heightened levels of career anxiety and indecision. For instance, recent empirical studies underscore that the pervasive culture of “involution” (neijuan) in China's highly competitive job market has triggered widespread somatic and career anxiety among university students (You et al., 2022; Yuan et al., 2025), making the investigation of psychosocial protective factors practically critical. Both countries are undergoing a dramatic transition characterized by the popularization of higher education and the modernization of their occupational structures, which presents unprecedented opportunities and challenges for their youth.

However, deep-seated differences in cultural foundations may be associated with divergences in the internal logic of career decision-making among the youth of the two countries. Chinese society is deeply associated with Confucian culture, which emphasizes family-centeredness and collective harmony. The career choices of young people often need to be embedded within the dual considerations of family expectations to “bring glory to the family” and the social evaluation of the “culture of ‘face”' (Zhu et al., 2023; Zhou et al., 2024). In contrast, the cultural landscape of Kazakhstan is more complex, blending the collectivism and tribal honor of traditional nomadic peoples with the modernizing demands of a rapid social transition following the dissolution of the Soviet Union. Consequently, the career choices of Kazakhstani youth may be constrained by traditional factors like “family reputation” and elder authority, while also facing a conceptual clash and tension between “traditional stable occupations” and “modern risk-taking occupations” (such as information technology, entrepreneurship) (Buribayev et al., 2025; Tolesh, 2022). This unique cultural heterogeneity provides a natural experimental context with distinct cultural contrast value for exploring how “family capital and cultural concepts jointly relate to an individual's career psychology.”

A review of existing research reveals its limitations in exploring such issues. On the one hand, the geographical scope of most cross-cultural vocational psychology studies focuses on the binary opposition between “Western individualism” and “East Asian collectivism,” with relatively limited attention to the key transitional region of Central Asia. Relying solely on this limited East-West binary literature underestimates the complex socio-cultural transition models of Central Asian countries, creating a significant conceptual gap. This study attempts to supplement the existing literature with a broader range of intercultural empirical comparisons. On the other hand, research methods often rely on participants' “retrospective reports” (e.g., directly asking “How was your family related to your career choice?”). While convenient, this method struggles to accurately capture the immediate, contextual associations of various family capitals and cultural concepts during the dynamic decision-making process.

Based on the above background, this study attempts to specifically address these two gaps: first, to extend the geographical scope of research from the traditional “West-East Asia” binary framework to a transitional Central Asian country, filling the research gap in this region with a direct empirical comparison between China and Kazakhstan; second, to replace retrospective reports with structured multi-dimensional scales to more accurately capture the immediate association of family capital and cultural concepts in the career decision-making process. On this basis, this study constructs a cross-cultural empirical interactive model that integrates family multidimensional socioeconomic status, cultural values, and career self-determination, aiming to precisely answer the following refined core questions:

1. To what extent does subjective social class perception mediate the associations between multidimensional family capital (economic, cultural, and social) and youth career self-determination?
2. Do traditional vs. modern cultural values serve as moderating boundary conditions within these socio-cognitive relationship paths?
3. How do these complex predictive mechanisms differ structurally between the contrasting socio-economic conditions of China and Kazakhstan?

This study not only hopes to enrich the applicability and explanatory power of self-determination theory in non-Western, especially transitional, country contexts, but also aims to provide precise intervention anchor points for the education departments of China and Kazakhstan to formulate and optimize youth career counseling policies by providing culturally sensitive empirical data.

## 2 Theoretical basis and research hypotheses

By explicitly linking Self-Determination Theory (SDT)—which emphasizes intrinsic psychological needs—with Social Cognitive Career Theory (SCCT)—which maps environmental resource affordances—this study establishes a rigorous conceptual clarity. Within this integrated framework, objective family capital acts as the SCCT environmental input, subjective class perception serves as the cognitive appraisal pathway, and career self-determination represents the ultimate SDT motivational outcome. Meanwhile, differing cultural contexts are posited to act as macro-level boundary conditions that shape these pathways.

### 2.1 Career self-determination: from theory to measurement

Self-Determination Theory (SDT) provides a key perspective for understanding the intrinsic motivation behind career choices. The theory posits that the motivation for human behavior can be placed on a continuum from external control to internal drive. When an individual's behavior stems more from internal interest and value identification, satisfying the basic psychological needs for Autonomy, Competence, and Relatedness, their behavior will be more sustainable and creative (Ryan et al., 2025).

In the career domain, this theory is associated with the development of “career self-determination,” which describes an individual's psychological capacity and state in the decision-making process, rather than a specific career preference. A high level of career self-determination is typically manifested in three measurable dimensions: First, career decision-making autonomy, the feeling that choices are made out of personal will rather than external pressure; second, career decision-making competence, the confidence in one's ability to make the right decisions; and third, career exploration behavior, the active initiative to understand oneself and the professional world (Fugate et al., 2004). A large body of research has confirmed that a high level of career self-determination is closely related to clearer career goals and stronger career adaptability in youth (Li et al., 2023; Lu et al., 2024). Therefore, exploring its correlates is of great value. Following the internal logic of self-determination theory, this study operationalizes “career decision-making autonomy” as a unified construct encompassing “freedom from external pressure” and “the pursuit of realizing intrinsic values” (Ryan and Deci, 2000).

### 2.2 The direct effect of family socioeconomic status

As the primary environment for an individual's growth, a family's socioeconomic status (SES) is associated with a child's career development through multidimensional capital. Bourdieu's (1986) theory of capital provides a comprehensive framework, identifying economic, cultural, social, and symbolic capital (prestige and honor). While symbolic capital is undoubtedly important, it is often intertwined with and expressed through the other three forms, making its independent measurement particularly complex in a cross-cultural context. Therefore, for the purpose of achieving a clear and empirically tractable comparison, this study deliberately focuses on the three more directly measurable forms of capital—economic, cultural, and social—to examine their specific association mechanisms in the career domain. These capitals collectively form the resource base for a young person's career choices: economic capital broadens the boundaries of choice, cultural capital enhances decision-making wisdom, and social capital provides direct opportunities (Lin, 2001; Yang and Su, 2025). Furthermore, psychological research complements this with the importance of subjective social status (Adler et al., 2000), which is an individual's subjective perception of their class position and is directly related to self-confidence and a sense of control. Taken together, a more advantageous family SES, whether in terms of objective resources or subjective feelings, should provide young people with stronger psychological support and a broader space for exploration, thereby being positively associated with the development of their career self-determination. Based on this, the study proposes the first main effect hypothesis:

H1: Family socioeconomic status (including objective capital and subjective class perception) is significantly positively correlated with the level of career self-determination in youth.

Social Cognitive Career Theory (SCCT) further points out that the association between environmental resources on an individual's career behavior often involves the intermediate level of psychological perception (Gheyassi and Alambeigi, 2024; Lent et al., 1994). The research by (Adler et al. (2000) also indicates that objective family capital conditions are not directly associated with an individual's psychological state and behavior, but are first internalized into the individual's subjective perception of their social position, which in turn is associated with their self-confidence, sense of control, and propensity for action. Kraus et al. (2012) further noted that subjective social class perception, as a psychological mapping of objective capital conditions, is an indispensable intermediate link in understanding the “resource → behavior” relationship.

Based on the above theories, this study proposes a mediation effect hypothesis:

H1a: Subjective social class perception mediates the relationship between family objective capital (economic capital, social capital) and career self-determination. Specifically, more abundant objective family capital is first associated with an individual's positive perception of their social status, and this positive class self-positioning is in turn positively associated with higher levels of career decision-making autonomy and exploration behavior.

### 2.3 The moderating role of cultural values

Cultural values, by shaping an individual's beliefs about the “ideal career,” may moderate the association with family resources (Hofstede, 2001; Tang et al., 2022). Under traditional cultural concepts that emphasize family interests and collective honor, an individual's choices are more easily constrained by family expectations, and the association of family resources may thus be amplified (Schwartz, 1992). The family-centered ideology in China and the sense of family honor in Kazakhstan both reflect a collectivist cultural background. Therefore, traditional cultural values may have a moderating association with the “family SES—career self-determination” relationship.

Accordingly, a moderation effect hypothesis is proposed:

H2: Traditional cultural values play a moderating association with the relationship between family SES and career self-determination. Specifically, this study proposes two competing predictions for the direction of moderation: (a) Inhibitory moderation: Among individuals with high traditional values, the positive association between family SES and career self-determination is weakened, because the pressure to conform brought by traditional norms may offset the promoting association of resource advantages on individual autonomy (based on SDT); (b) Amplifying moderation: Among individuals with high traditional values, the positive association between family SES and career self-determination is enhanced, because high-SES families may provide stronger shelter and support for their children within a traditional collectivist framework (based on Schwartz, 1992). This study will test these two possibilities with empirical data.

### 2.4 Cross-cultural heterogeneity: the specific impact of national conditions in China and Kazakhstan

Although family capital and cultural concepts have a universal association, their specific association mechanisms are likely to vary depending on national conditions. In China's highly competitive social environment, the “economic threshold” for education and employment is increasingly prominent, making economic capital possibly the most critical factor associated with the space for career choices (Liu and Zang, 2024). In contrast, in Kazakhstan, where the social structure retains strong traditional ties, social capital (interpersonal networks) originating from family and community may have a more central association with career recommendations and patronage (Amankulova, 2024; Khussainova et al., 2023). This difference in social structure may be associated with different dimensions of family capital having different weights of association with the youth of the two countries. Therefore, the core cross-cultural hypotheses of this study are as follows:

H3: In the Chinese sample, the positive association of family economic capital on career self-determination will be significantly stronger than its association in the Kazakhstani sample.

H4: In the Kazakhstani sample, the strength of the association between family social capital and career self-determination will be significantly higher than in the Chinese sample.

**Figure 1:**
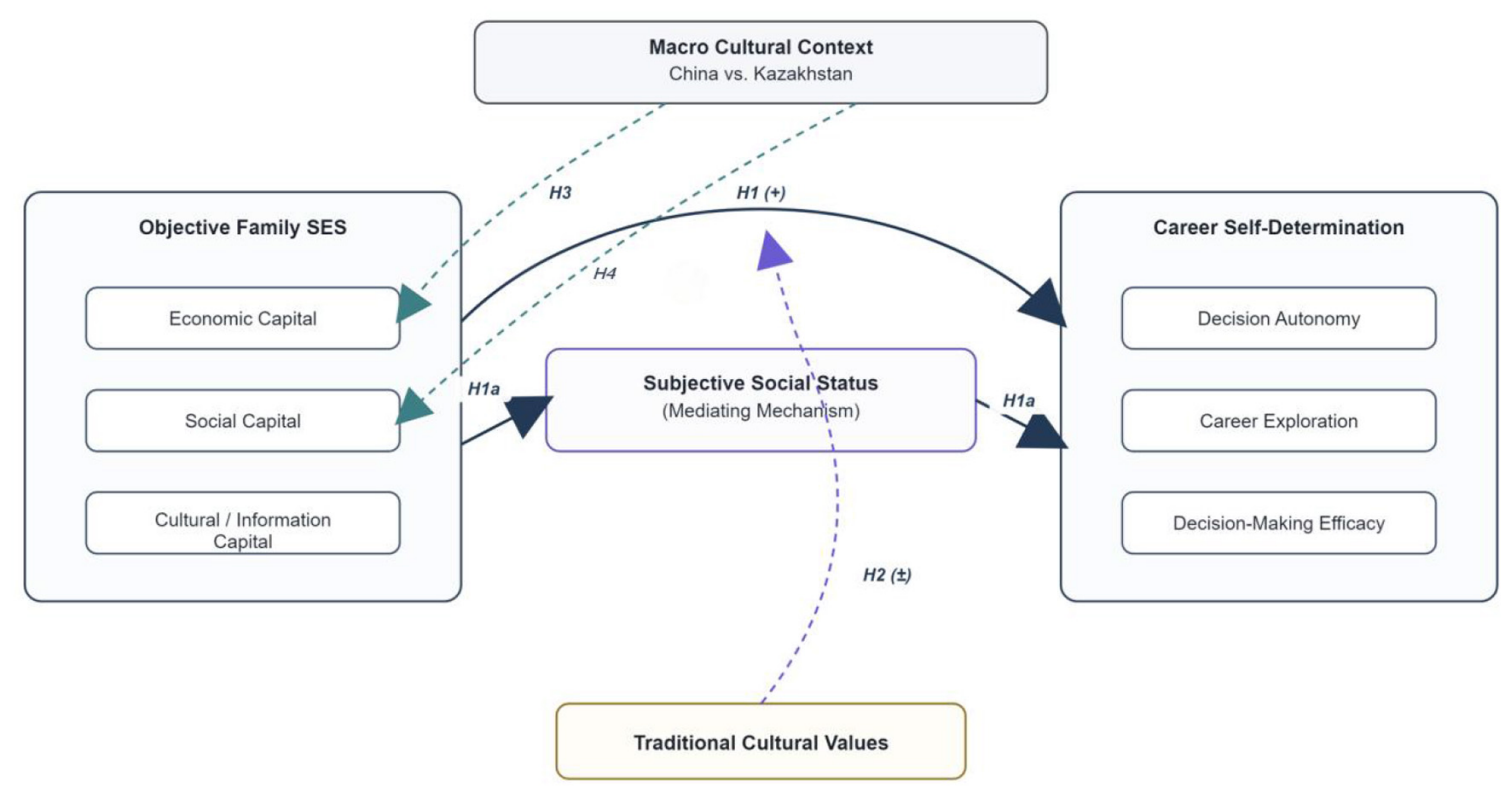
The proposed theoretical model. Solid arrows represent direct and mediating associations. Dashed arrows represent moderating associations, indicating that the moderating variable (e.g., Macro Cultural Context) is hypothesized to alter the strength of the path it points to.

By testing the above hypotheses, this study aims to construct a comprehensive model that integrates family socioeconomic status, cultural values, and cross-national contexts to reveal the internal relational patterns of youth career development in different socio-cultural ecologies. The overall theoretical framework is shown in Figure 1.

## 3 Research methods

### 3.1 Participants and sampling

This study focused on a specific demographic defined as “youth.” For the purpose of this study, “youth” are university students aged 18 and above, who are at a critical stage of career decision-making. This study employed a cross-cultural cross-sectional survey method. Initially, a total of 1300 questionnaires were collected through various online channels in China and Kazakhstan. To ensure data quality and the rigor of research ethics, the researchers implemented a strict dual cleaning protocol on the raw data: Based on explicit inclusion and exclusion criteria. The inclusion criteria required that participants be currently enrolled students in higher education (university or vocational colleges) and possess independent civil capacity (aged 18 or above). The exclusion criteria consisted of: (1) all invalid samples from participants under the age of 18; (2) incomplete questionnaires missing core variable data; (3) invalid questionnaires showing straight-lining (i.e., selecting the same option for all scored items, *SD* = 0). After screening, a final total of 1020 valid questionnaires were retained, resulting in an effective response rate of 78.46%.

### 3.2 Instrumentation

All measurement tools in this study were adapted or extracted from widely validated classic scales, and a translation-back-translation procedure was performed by psychology professionals from both China and Kazakhstan to safeguard conceptual equivalence in the cross-cultural context. The detailed questionnaire can be found in the Supplementary materials.

Family Socioeconomic Status (SES): A composite measurement method was used. The objective indicators section included parents' highest level of education and monthly family income, and borrowing from the core dimensions of the “Parental Career Support Behavior Scale” by Dietrich and Kracke (2009), it measured specific support behaviors from the family in three dimensions: economic, informational, and network-related. The subjective indicators section used the “MacArthur Scale of Subjective Social Status” by Adler et al. (2000) to measure an individual's self-perception on the social ladder.

Cultural Values: Core items were adapted from the “Vertical Collectivism Scale” by Triandis and Gelfand (1998) and related research. “Traditional cultural values” were measured with 4 items (e.g., “In career choices, I should prioritize the overall interests of my family”); “Modern individualistic tendencies” were assessed with a single exploratory item (“I believe the standard of career success should absolutely be defined by myself…”).

Career Self-Determination: To provide a comprehensive assessment encompassing motivational (autonomy), behavioral (exploration), and cognitive (efficacy) components, this construct was operationalized as a multi-dimensional composite indicator (11 items). Items were assessed using a 5-point Likert scale (1 = *strongly disagree*, 5 = *strongly agree*), with higher scores indicating stronger career agency.

Career Decision-Making Autonomy (Autonomy): Based on Guay's (2005) “Career Decision-Making Autonomy Scale” (CDMAS), and incorporating the emphasis on intrinsic value orientation of “autonomy” in Self-Determination Theory (SDT), this study integrated the core items of the original scale with two items in the questionnaire about “intrinsic career motivation” (e.g., “Compared to salary and status, I value enthusiasm for the work more”). Factor analysis results clearly indicated that these 5 items collectively pointed to a single, highly homogeneous latent factor. Therefore, they were merged to measure “career decision-making autonomy,” which not only enhanced the reliability of the scale but also more closely aligned with the complete connotation of the theoretical construct.

Career Exploration Behavior (Exploration): This dimension (3 items) was adapted from the classic “Career Exploration Survey” (CES) by Stumpf et al. (1983) to assess an individual's specific actions in actively understanding themselves and the professional world. (e.g., “I proactively read books, browse industry websites, or attend courses to gain a deeper understanding of the real work status of different professions”).

Career Decision-Making Efficacy (Efficacy): This dimension (3 items) used the “Career Decision-Making Self-Efficacy Scale-Short Form” (CDMSE-SF) by Betz et al. (1996) to measure an individual's confidence and sense of efficacy when facing career decisions. (e.g., “When faced with multiple tempting or risky career options, I am capable of making a clear decision that I will not regret”).

### 3.3 Data collection and ethical considerations

The research protocol was approved by the Institutional Review Board (IRB) of the collaborating institutions. The questionnaire was distributed through online platforms (e.g., WJX.cn in China and Google Forms in Kazakhstan), and disseminated through institutional email lists and student forums at the collaborating universities. All participants were required to read and agree to the informed consent form before starting the questionnaire, which explicitly stated the anonymity of the study, data confidentiality principles, and the right of participants to withdraw unconditionally at any time.

### 3.4 Data analysis strategy

This study used SPSS 27.0, AMOS 26.0, and the R Programming language for data processing and hypothesis testing. The analysis steps were as follows:

Data Quality Control: Performed data cleaning and used Harman's single-factor test to assess common method bias.

Descriptive Statistics and Difference Testing: Used means and standard deviations to present an overview of the data, and independent samples t-tests to compare baseline differences in core variables between the Chinese and Kazakhstani samples.

Reliability and Validity Testing: Used Cronbach's α, Composite Reliability (CR), and Average Variance Extracted (AVE) to assess the reliability and validity of the measurement tools.

Correlation Analysis: Used Pearson correlation coefficients to explore the initial associations between variables (Harman, 1976).

Moderation Effect Test: Used the PROCESS macro (Model 1) to test the moderating association of traditional cultural values.

Mediation Effect Test: Used the bias-corrected bootstrap procedure (PROCESS macro Model 4, 5000 resamples), with subjective social class perception as the mediator, family economic capital and social capital as independent variables respectively, and career self-determination as the outcome variable, to test the mediation association proposed in H1a, and reported the 95% confidence interval of the indirect effect. When the confidence interval did not include zero, the mediation association was considered significant.

Cross-cultural Multi-group Structural Equation Model Analysis (Multi-group SEM): After testing for measurement invariance, the core cross-cultural hypotheses were tested by comparing the group differences in path coefficients (χ^2^ difference test).

Relative Weight Analysis (RWA): Used the R language to precisely decompose the relative importance of different family capital dimensions in their association with self-determination, to overcome the problem of multicollinearity.

## 4 Research results

### 4.1 Measurement model and quality check

Before conducting the core hypothesis tests, this study first rigorously evaluated the reliability, validity, and potential biases of all measurement tools to check the reliability and validity of subsequent analyses.

#### 4.1.1 Common method bias test

To assess potential systematic errors in the self-report questionnaire, this study used a combination of exploratory and confirmatory approaches for bias testing. First, Harman's single-factor test was applied to all core scale items through an unrotated factor analysis. The results showed that the first principal component explained 42.11% of the total variance, which is below the critical threshold of 50%.

To further enhance the rigor of the test, this study used Confirmatory Factor Analysis (CFA) to compare the fit of a single-factor model (in which all latent variable measurement items are forced to load onto one latent factor) with the expected multi-factor measurement model. The results showed that the fit indices of the single-factor model were extremely poor (CFI = 0.826, TLI = 0.791, RMSEA = 0.144), significantly worse than the expected theoretical measurement model, and the chi-square difference test was significant (*p* < 0.001). The aforementioned multidimensional evidence collectively confirmed that the data in this study do not have serious common method bias.

#### 4.1.2 Reliability, validity, and measurement model fit

The measurements of each core latent variable in this study demonstrated good reliability and validity. As shown in Table 1, the Cronbach's α coefficients for all dimensions were above 0.78, composite reliability (CR) values were all higher than 0.70, and average variance extracted (AVE) values were all above the recommended statistical standard of 0.50, indicating that the measurement tools have excellent internal consistency and convergent validity.

**Table 1:** Reliability and convergent validity test of core latent variables.

Latent variable	No. of items	Cronbach's α	Composite reliability (CR)	Average variance extracted (AVE)
Traditional cultural values (tradition)	4	0.78	0.84	0.57
Career decision-making autonomy (autonomy)	5	0.83	0.85	0.65
Career exploration behavior (exploration)	3	0.75	0.79	0.56
Decision-making efficacy (efficacy)	3	0.81	0.77	0.52

In terms of discriminant validity, as shown in Table 2, a test using the Fornell-Larcker criterion found that the correlation coefficients between the three sub-dimensions of “Career Decision-Making Autonomy,” “Exploration Behavior,” and “Efficacy” (ranging from 0.812 to 0.826) were all higher than the square root of their respective AVEs (Fornell and Larcker, 1981). According to the Fornell-Larcker criterion, this indicates insufficient discriminant validity among the three sub-dimensions, meaning that the variance they jointly measure is greater than the variance they each measure independently. The high correlation among these three sub-dimensions (*r* = 0.812 to 0.826), and their theoretical representation of ‘an individual's psychological autonomous state in career decision-making,' together suggest the existence of a higher-order factor structure. To systematically address the issue of insufficient discriminant validity while retaining the independent measurement information of each dimension, this study used a second-order confirmatory factor analysis (Second-order CFA) to model career self-determination: Career Decision-Making Autonomy, Career Exploration Behavior, and Career Decision-Making Efficacy were set as three independent first-order factors, and the higher-level psychological construct they all point to was set as the second-order factor ‘Career Self-Determination (CSD)'.

**Table 2:** Discriminant validity test of core latent variables (Fornell-Larcker criterion).

Latent variable dimension	1. Tradition	2. Autonomy	3. Exploration	4. Efficacy
1. Traditional cultural values (tradition)	(0.755)			
2. Career decision-making autonomy (autonomy)	−0.409	(0.806)		
3. Career exploration behavior (exploration)	−0.387	0.826	(0.748)	
4. Decision-making efficacy (efficacy)	−0.418	0.821	0.812	(0.721)

Values in parentheses on the diagonal are the square roots of the AVE for each latent variable. Off-diagonal values are the correlation coefficients between latent variables.

The results of the second-order CFA showed that the model fit was good overall (CFI = 0.999, TLI = 0.998, RMSEA = 0.016), and the loadings of the three first-order factors on the second-order factor were all high and significant (Autonomy: λ = 1.00; Exploration: λ = 0.94; Efficacy: λ = 0.90, all *p* < 0.001), supporting the reasonableness of career self-determination as a unified higher-order construct.

The advantage of this modeling approach is that it captures the common variance of the three dimensions through the higher-order factor, while preserving the independent measurement structure of each first-order factor. This allows career self-determination to be used as an overall higher-order construct in main effect and cross-cultural SEM analyses, while the observed data of each sub-dimension can be used independently for descriptive reporting and correlation analysis.

Finally, the baseline multi-factor measurement model, verified by CFA, exhibited excellent construct validity (CFI = 0.996, TLI = 0.995, RMSEA = 0.021), providing a solid foundation for hypothesis testing.

Finally, the baseline multi-factor measurement model constructed in this study was validated by CFA and demonstrated excellent construct validity. The model's overall goodness-of-fit was excellent (CFI = 0.996, TLI = 0.995, RMSEA = 0.021), meeting and in some cases exceeding the thresholds conventionally recommended by scholars. This proves that the measurement tools of this study are of reliable quality, providing a solid foundation for subsequent hypothesis testing.

### 4.2 Descriptive statistics and correlation analysis

This study ultimately retained 1,020 valid questionnaires, with 524 from the Chinese sample (51.4%) and 496 from the Kazakhstani sample (48.6%), indicating a balanced distribution of sample sizes between the two countries. The specific socio-demographic characteristics of the sample are detailed in Table 3.

**Table 3:** Description of sample demographics.

Demographic variable	Category	Combined sample (*N* = 1,020) (%)	Chinese sample (*n* = 524) (%)	Kazakhstani sample (*n* = 496) (%)
Gender	Male	512 (50.2%)	261 (49.8%)	251 (50.6%)
	Female	508 (49.8%)	263 (50.2%)	245 (49.4%)
Age		21.60 ± 2.38 years	21.60 ± 2.38 years	21.59 ± 2.38 years
Current educational stage	Early university (Freshman/Sophomore)	108 (10.6%)	52 (9.9%)	56 (11.3%)
	Late university (junior/senior)	533 (52.3%)	279 (53.2%)	254 (51.2%)
	Vocational and technical college	281 (27.5%)	147 (28.1%)	134 (27.0%)
	Master's degree or above	98 (9.6%)	46 (8.8%)	52 (10.5%)
Long-term place of origin	Tier-1/core cities	331 (32.5%)	202 (38.5%)	129 (26.0%)
	Tier-2/3/smaller cities	472 (46.3%)	216 (41.2%)	256 (51.6%)
	County/towns and rural areas	217 (21.3%)	106 (20.2%)	111 (22.4%)

As shown in Table 3, the respondent sample demonstrated high cross-national consistency and internal balance in terms of gender ratio and age structure. The average age of the entire sample was approximately 21.60 years, and in terms of educational stage, over 80% of individuals were in their later years of university or at a vocational technical college. This demographic distribution highly aligns with the research objectives of this study, accurately targeting the youth group facing actual job-seeking pressure and career choice crossroads, providing an ideal empirical field for observing their career self-determination status and its antecedent variables.

#### 4.2.1 Cross-national baseline differences in core variables

**Figure 2:**
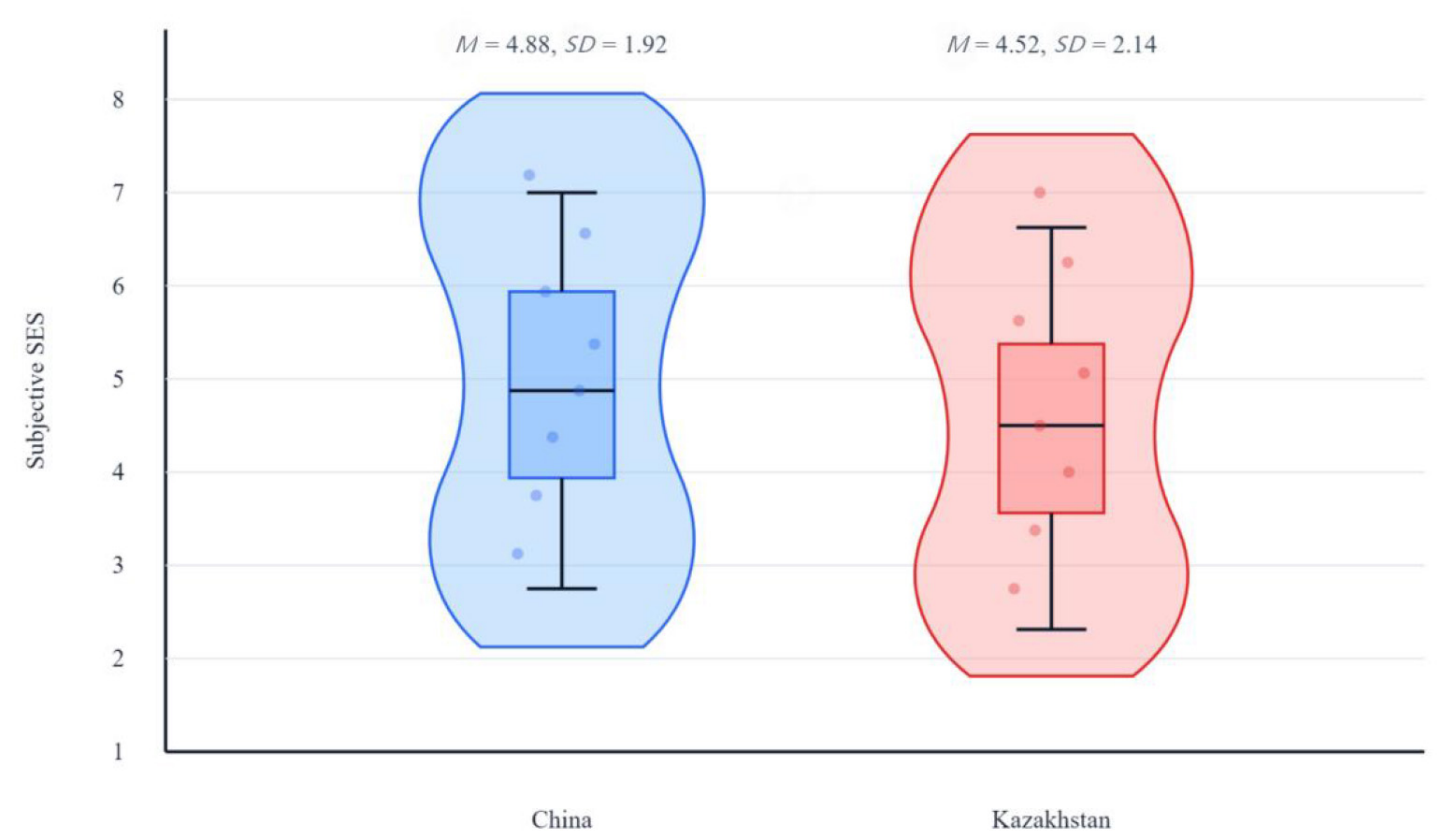
Comparison of subjective social status distribution between Chinese and Kazakhstani youth.

To explore the cross-national baseline differences in cultural perceptions and family capital perception between Chinese and Kazakhstani youth, this study performed independent samples t-tests on each core variable. As shown in Table 4, the Chinese youth respondents reported significantly higher subjective social status (*t* = 2.51*, p* < 0.05) and family economic support levels (*t* = 2.22*, p* < 0.05) than the Kazakhstani youth. The specific distribution patterns and differences in subjective social status perception between the two groups are shown in Figure 2. However, in terms of endorsement of traditional cultural values, modern individualistic values, and family social network support, the two national samples did not show statistically significant differences (all *p* > 0.05).

**Table 4:** Cross-national independent samples *t*-test of core variables.

Latent variable name	Chinese sample (*n* = 524) *M* (*SD*)	Kazakhstani sample (*n* = 496) *M* (*SD*)	*t-*value	*p-*value
Subjective social status perception	5.01 (2.31)	4.65 (2.25)	2.51^*^	0.012
Family economic capital support	3.07 (0.97)	2.94 (0.99)	2.22^*^	0.027
Family social network capital	2.98 (1.01)	2.90 (1.01)	1.39	0.165
Traditional cultural values	3.36 (0.81)	3.33 (0.82)	0.57	0.566
Modern cultural values	3.75 (0.97)	3.72 (0.93)	0.51	0.609

^*^p < 0.05.

This comparison reveals that while both groups perceive similar levels of social network access and hold similar cultural values on average, Chinese students perceive their families as having a higher socioeconomic standing, both subjectively and in terms of economic support.

#### 4.2.2 Correlation analysis of core variables

After verifying the basic distribution characteristics of the variables, this study used Pearson correlation coefficients to preliminarily explore the direction and strength of the associations between the core variables. As shown in Table 5, the results indicate that subjective social status, economic capital, and social capital were all significantly positively correlated with the total score of career self-determination (*r* = 0.43, 0.35, and 0.34 respectively, representing medium to strong effect sizes (Cohen, 1988), all *p* < 0.001) supporting the basic theoretical framework of the study. Meanwhile, traditional cultural values were significantly negatively correlated with career self-determination (*r* = −0.43*, p* < 0.001), while modern values were positively correlated (*r* = 0.39*, p* < 0.001), providing preliminary evidence for the subsequent moderation effect analysis.

**Table 5:** Correlation matrix of core variables.

Variable	1	2	3	4	5	6
1. Subjective status perception	1					
2. Economic capital support	0.52^***^	1				
3. Social network capital	0.35^***^	0.05	1			
4. Traditional cultural values	0.03	−0.02	0.01	1		
5. Modern individualistic values	0.04	−0.01	0.05	−0.05	1	
6. Career self-determination	0.43^***^	0.35^***^	0.34^***^	−0.43^***^	0.39^***^	1

^***^p < 0.001.

**Figure 3:**
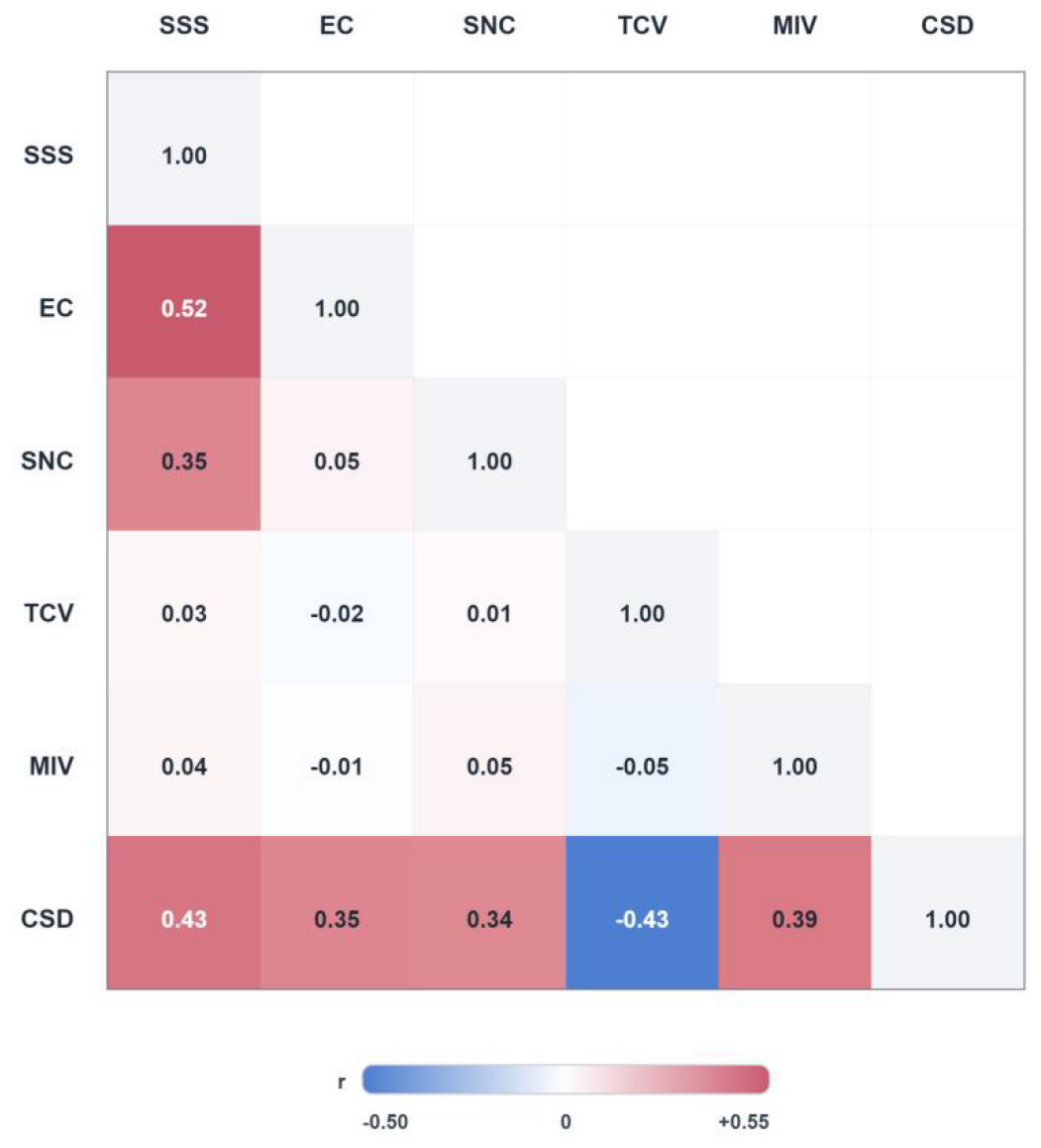
Heatmap of core variable correlations. The figure shows the Pearson correlation matrix of the core variables. The color intensity represents the correlation strength; red indicates a positive correlation, and blue indicates a negative correlation. SSS, Subjective Social Status; EC, Economic Capital; SNC, Social Network Capital; TCV, Traditional Cultural Values; MIV, Modern Individualistic Values; CSD, Career Self-Determination.

To further visually present the overall association patterns and strength distribution among the above core variables, this study plotted a corresponding correlation visualization matrix as shown in Figure 3.

### 4.3 Main effect and moderation effect analysis

This study used hierarchical regression to test the main and moderation effects. For the structural simplicity of the main and moderation effect analysis, the hierarchical regression model here first used “subjective social status perception” as a comprehensive proxy for family socioeconomic status (SES) at the individual psychological level. As shown in Table 6, Model 1 shows that after controlling for demographic variables, subjective social status (SES) has a significant positive association with career self-determination (β = 0.449, *p* < 0.001), which provides preliminary core empirical support for H1. Traditional cultural values had a significant negative association with career self-determination (β = −0.446*, p* < 0.001).

**Table 6:** Hierarchical regression analysis of subjective social status and traditional values on career self-determination.

Predictor	Model 1 (β)	Model 2 (β)
Main effect	
Subjective social status (SES)	0.449^***^	0.449^***^
Traditional cultural values (tradition)	−0.446^***^	−0.446^***^
Interaction effect	
SES × tradition		0.024
*R* ^2^	0.380	0.380
Δ*R*^2^		0.000
*F* value	311.52^***^	207.61^***^

β is the standardized regression coefficient.

^***^p < 0.001.

Model 2 added the interaction term between SES and traditional values to test the moderating association (H2). The results showed that the coefficient of the interaction term was not significant (β = 0.024, *p* = 0.343), and there was no significant change in ΔR^2^, indicating that in the combined sample, the moderating association of traditional cultural values on the “SES-career self-determination” relationship was not supported, that is, H2 was not confirmed. For the sake of rigor, to rule out the possibility of a statistical cancellation effect in the combined sample due to different cultural operating logics in China and Kazakhstan (e.g., positive moderation in one country and negative in the other), this study further conducted independent moderation association regressions on the Chinese and Kazakhstani sub-samples. The results showed that the interaction term was not significant within either country (Chinese sample interaction term: β = 0.012*, p* =0.735; Kazakhstani sample interaction term: β = 0.016, *p* = 0.657).

This cross-nationally consistent result from independent tests robustly indicates that the negative association of traditional values with career decision-making autonomy is universally prevalent across social classes, rather than only being significant within a specific social stratum.

**Figure 4:**
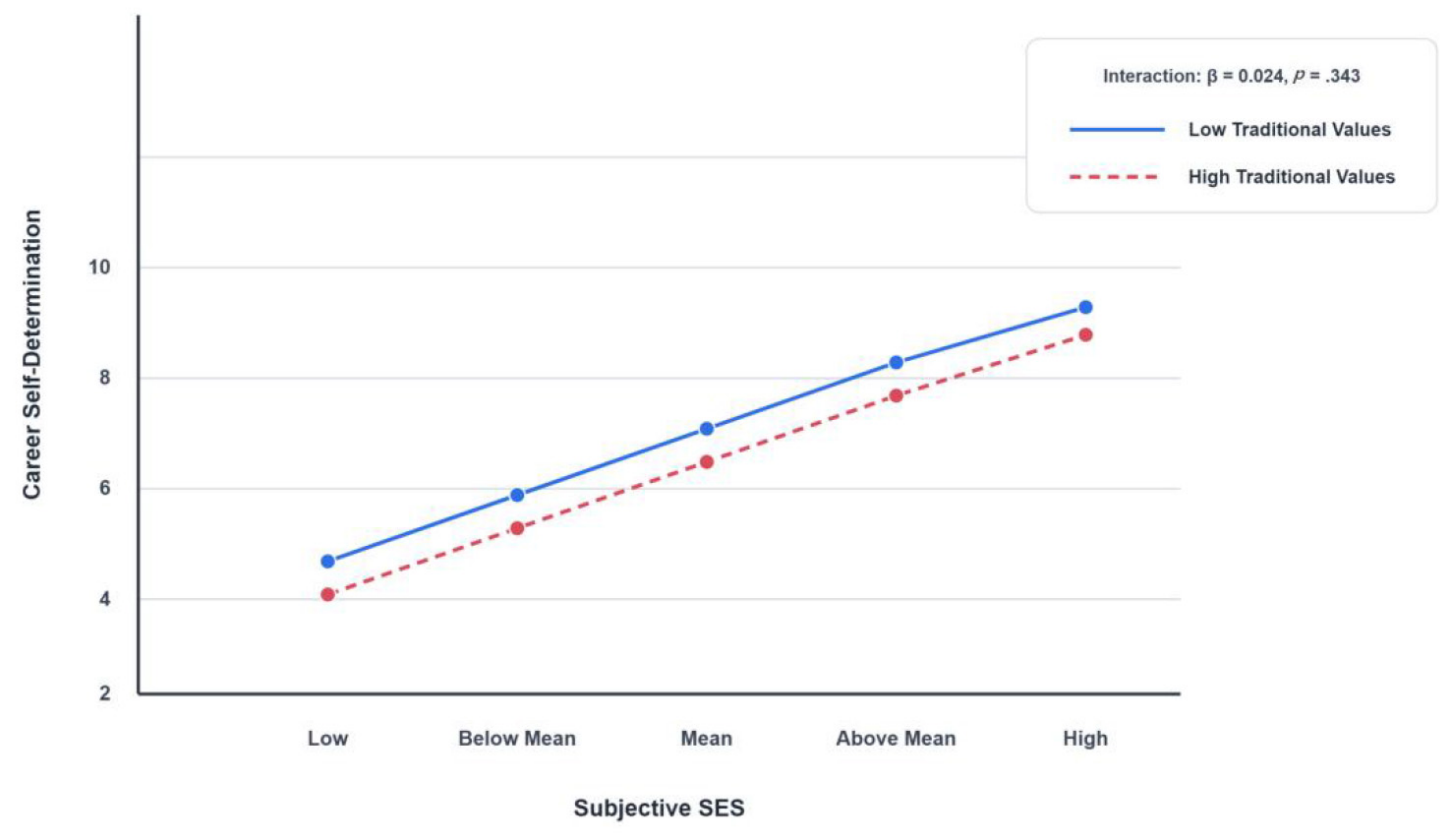
Simple slopes plot of the moderating association of traditional cultural values.

The non-significant interaction is visually represented in the simple slopes plot in Figure 4.

Although the interaction term was not significant, the simple slopes plot (see Figure 4) visually reveals the trend: For individuals who highly endorse traditional values (red dashed line), their level of career self-determination is generally lower than those with low traditional values (blue solid line). As family social status increases, the gap between the two slightly narrows, but the positive association with social status is not completely suppressed by traditional values.

### 4.4 Mediation effect test of subjective social status perception

After establishing the main effect of family SES, this study further tested H1a, which is whether subjective social status perception mediates the relationship between family objective capital (economic capital, social capital) and career self-determination. The theoretical logic of this hypothesis is that objective family capital conditions do not directly “exert force” on an individual's career psychology. Instead, they are first internalized into the individual's perception and evaluation of their social position, which in turn is associated with the autonomy and exploration behavior in their career decision-making through this subjective perception (Adler et al., 2000; Kraus et al., 2012; Wang et al., 2025).

The study used the bias-corrected bootstrap procedure (Model 4, 5,000 resamples) to assess the mediation association, running two parallel mediation models with economic capital and social capital as independent variables, respectively.

As shown in Table 7, in the mediation model with economic capital as the independent variable, family economic capital had a significant positive predictive association with subjective status perception (path a: β = 0.519*, t* = 19.40*, p* < 0.001); subjective status perception also had a significant positive predictive association with career self-determination (path b: β = 0.347, *t* = 10.62, *p* < 0.001); after including subjective status perception, the direct association of economic capital on career self-determination (path c': β = 0.169*, p* < 0.001) decreased compared to the total association (path c: β = 0.349). The Bootstrap analysis showed that the indirect association of economic capital on career self-determination through subjective status perception was 0.180 [95% CI (0.142, 0.219)], with the confidence interval not including zero, indicating a significant mediation association.

**Table 7:** Mediation association of subjective social status perception between family objective capital and career self-determination.

Model Path	Model A: economic capital as IV			Model B: social capital as IV		
	β	*t*-value	95% CI	β	*t*-value	95% CI
Path c (total effect X → Y)	0.349	11.88	(0.291, 0.407)	0.337	11.42	(0.279, 0.395)
Path a (X → subjective status)	0.519	19.40	(0.467, 0.571)	0.346	11.75	(0.288, 0.404)
Path b (subjective status → Y)	0.347	10.62	(0.283, 0.411)	0.361	12.30	(0.303, 0.418)
Path c' (direct effect)	0.169	5.18	(0.105, 0.233)	0.212	7.23	(0.155, 0.269)
a × b (indirect effect)	0.180	—	(0.142, 0.219)	0.125	—	(0.097, 0.154)

X, Independent Variable (Economic Capital for Model A, Social Capital for Model B); Y, Career Self-Determination; Bootstrap resampling 5,000 times.

In the mediation model with social capital as the independent variable, the association of family social capital on subjective status perception was also significant (path a: β = 0.346*, t* = 11.75*, p* < 0.001); path b for subjective status perception remained significant (β = 0.361*, t* = 12.30*, p* < 0.001). The Bootstrap indirect effect was 0.125 [95% CI (0.097, 0.154)], with the confidence interval not including zero, indicating a significant mediation effect.

**Figure 5:**
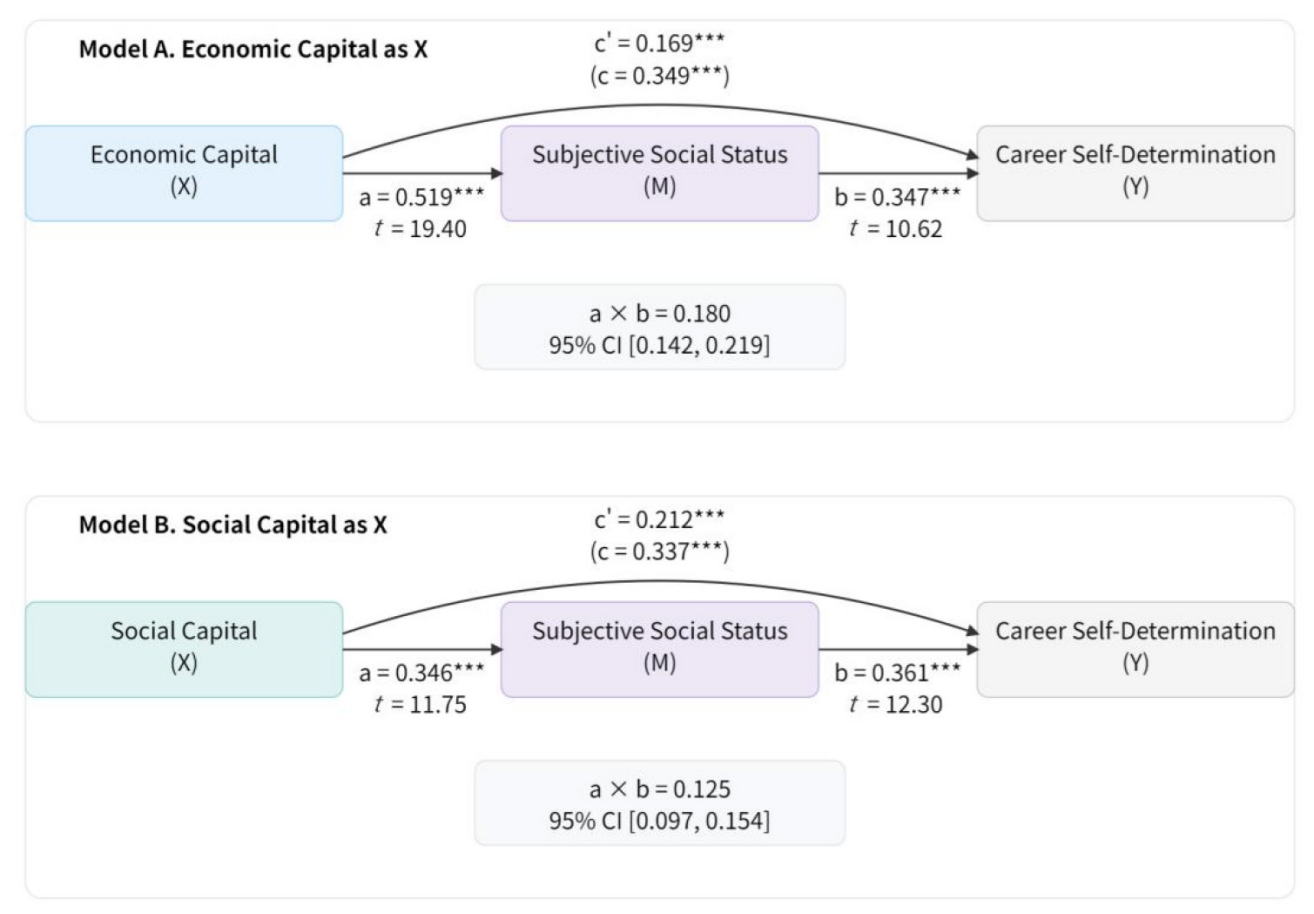
Mediation path diagram of subjective status perception. Model A uses economic capital as the independent variable, Model B uses social capital as the independent variable. The figure reports standardized path coefficients, direct effects, and indirect effects.

The above results indicate that in the two sets of models with economic capital and social capital as independent variables respectively, subjective social status perception demonstrated a stable mediating association. To more intuitively display this mediation mechanism, this study further drew a mediation path diagram based on the results of the bias-corrected bootstrap analysis. As shown in Figure 5, both sets of mediation models present a clear “Objective Capital—Subjective Status Perception—Career Self-Determination” transmission structure.

The results indicate that family objective capital not only has a direct positive association with career self-determination but also has an indirect association with career self-determination through the indirect association through subjective social status perception, supporting H1a. This finding is highly consistent with the theoretical framework of Kraus et al. (2012) regarding “objective resources being associated with individual individual psychology and behavior after being internalized through class perception”: An objectively advantageous family environment first associated with a more positive class self-positioning for the individual. This subjective perception of “I am in a higher position in society” in turn is associated with a stronger sense of autonomous will and exploratory drive in career decision-making.

### 4.5 Cross-cultural differences: multi-group structural equation modeling (multi-group SEM)

To deeply explore the heterogeneity of the association mechanism of family capital's role in different cultural backgrounds, this study constructed a multi-group structural equation model. First, the measurement invariance test indicated that the model was comparable between the Chinese and Kazakhstani samples. The configural invariance model showed a good fit (CFI = 0.999, RMSEA = 0.015); after imposing metric invariance constraints on the basis of configural invariance, the change in model fit did not exceed the critical value (ΔCFI = 0.001, ΔRMSEA = 0.002), meeting the requirements for metric invariance and supporting direct comparison between the cross-national samples; in the scalar invariance model (ΔCFI <0.01, supporting scalar invariance, allowing direct comparison of means across nations).

As shown in Table 8, the cross-national differences in key paths were extremely significant. In the Chinese sample, the path coefficient from economic capital to career self-determination (β = 0.50) was much larger than that of social capital (β = 0.10). In the Kazakhstani sample, the situation was completely reversed, with the path coefficient of social capital (β = 0.52) being much larger than that of economic capital (β = 0.18). The chi-square difference test showed that the cross-national differences in these two core paths reached a statistically significant level (*p* < 0.001), providing strong empirical support for H3 and H4.

**Table 8:** Comparison of differences in the “family capital → career self-determination” structural paths between China and Kazakhstan.

Structural path	Chinese sample (*n* = 524) standardized path coefficient (β)	Kazakhstani sample (*n* = 496) standardized path coefficient (β)	Group difference test (Δ χ^2^)
Economic capital → self-determination	0.50^***^	0.18^*^	15.23^***^
Social capital → self-determination	0.10 ns	0.52^***^	21.09^***^

ns indicates not significant.

^*^p < 0.05, ^***^p < 0.001.

### 4.6 Relative Weight Analysis

To move beyond the comprehensive proxy of subjective class perception and precisely quantify the independent contributions of the three objective family capital dimensions (economic, cultural, and social), this study employed Relative Weight Analysis (RWA). This method overcomes the issue of multicollinearity among the predictors and allows for a systematic decomposition of the explained variance (*R*^2^).

As shown in Table 9, the results clearly reveal a fundamental difference in the types of resources that the youth of the two countries rely on for career decision-making. In China, economic capital (72.4%) holds an absolutely dominant position in predicting career self-determination. In Kazakhstan, social network capital (69.1%) becomes the most important predictor. The contribution of informational/cultural capital is relatively stable in both countries (about 26%), indicating that the transmission of knowledge and information is an important support factor in different cultures.

**Table 9:** Relative contribution of family capital dimensions to career self-determination (%).

Capital dimension	Chinese sample (%)	Kazakhstani sample (%)
Economic capital (economy)	72.4	5.1
Informational/cultural capital (info)	26.1	25.8
Social network capital (social)	1.5	69.1
Total	100.0	100.0

**Figure 6:**
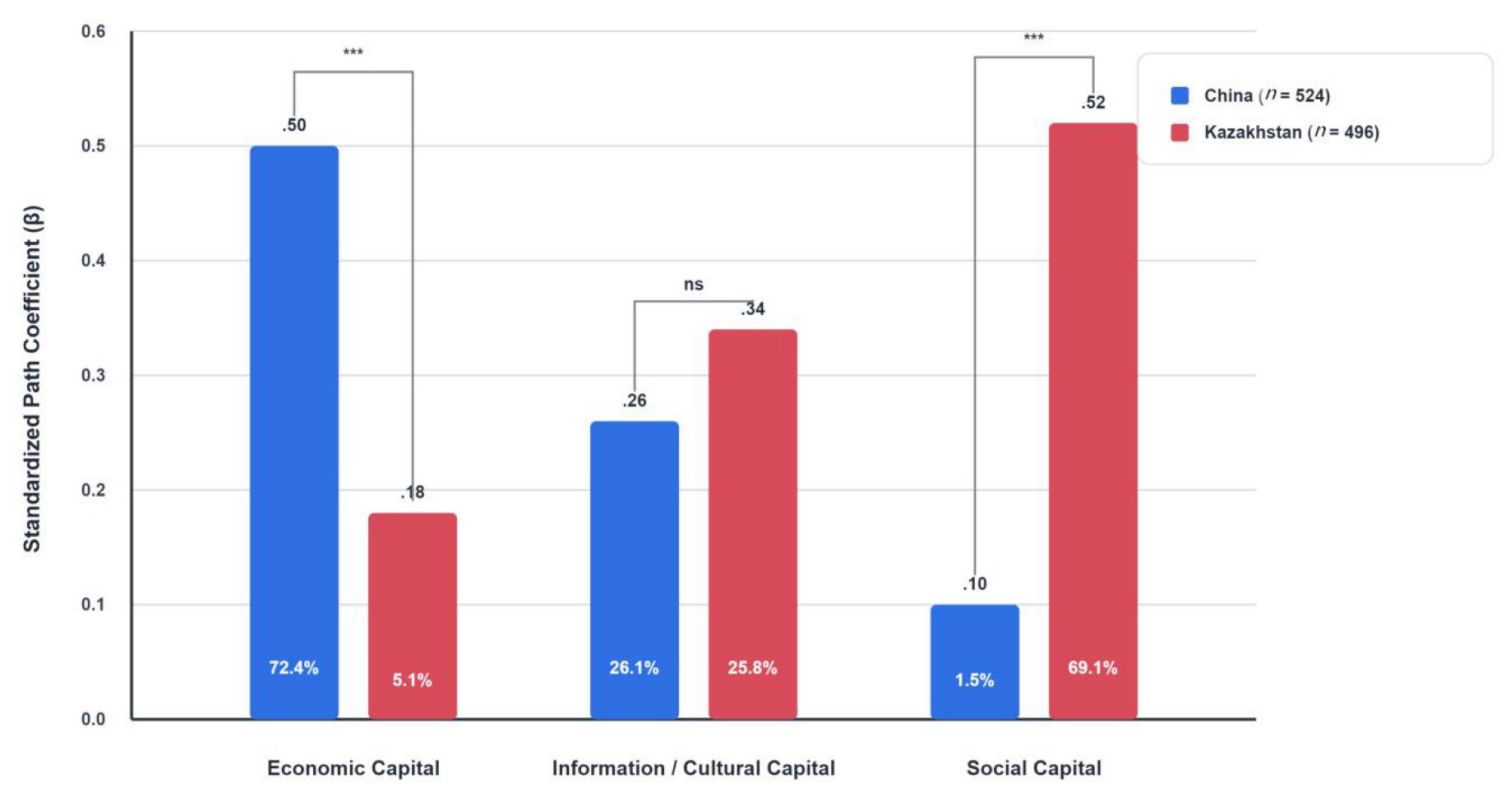
A cross-national comparison of the associations of family capital on career self-determination. The values on top of the bars are standardized path coefficients (β). The percentages inside the bars are the contribution proportions obtained from Relative Weight Analysis (RWA). Blue represents the Chinese sample, red represents the Kazakhstani sample. ***p < 0.001; ns, not significant.

Figure 6 visually summarizes these cross-national differences by presenting the standardized path coefficients alongside their corresponding relative weights. The graphical patterns clearly illustrate the contrasting reliance on economic capital for Chinese students and social network capital for Kazakhstani students.

It is noteworthy that the results in Table 4 show no significant difference in the mean of family social network capital between the two national samples (*t* = 1.39*, p* = *0.1*65), while the RWA results here show that the relative contribution weight of social capital in Kazakhstan is as high as 69.1%. These two findings are not statistically contradictory: The independent samples *t*-test compares the perceived quantity level of social capital by the youth of the two countries, whereas the RWA weight reflects the strength of the association between social capital and career self-determination and its relative importance among all predictor variables.

## 5 Discussion

### 5.1 The association mechanism of family capital on career self-determination

The results of this study first confirm the universal importance of family socioeconomic status as a key variable associated with youth career development. Both subjective class perception and objective capital support are significantly positively associated with various dimensions of career self-determination (autonomy, exploration behavior, competence), which is consistent with the conclusions of previous research (Wang et al., 2025; Wang and Dong, 2024). This aligns with the theoretical assumption that family capital serves as an important psychological buffer and practical support (Bourdieu, 1986), but the causal direction awaits further clarification from longitudinal studies.

This study further reveals a noteworthy association path: the association between family objective capital and career self-determination appears to operate partially through the psychological mechanism of subjective social class perception. The Bootstrap mediation analysis shows that both economic capital and social capital have a significant indirect association with career self-determination through subjective social class perception, supporting H1a. This finding is highly consistent with the theoretical assertion by Kraus et al. (2012) that class perception is a key psychological mediator between resources and behavior. This critical analytical finding deepens existing literature (Wang et al., 2025) by demonstrating that objective resource discrepancies do not mechanically determine psychological outcomes; rather, cognitive self-appraisal is the necessary catalyst. This aligns seamlessly with recent advancements in vocational psychology, which assert that objective resources—such as family wealth or study affordances—can only facilitate career optimism and exploratory drive once they are subjectively appraised by students as meaningful psychological capital (Nerona et al., 2025). This challenges purely materialistic sociological views and asserts the vital role of cognitive psychology in career trajectories. It reveals a specific psychological association pathway whereby objectively superior family capital conditions do not directly “endow” youth with stronger career decision-making ability, but instead first help youth form an internal perception of “I am in a good position in society.” This positive self-positioning, in turn, activates a stronger autonomous will and exploratory drive in career choices. In other words, the “material advantage” of the family must be internalized by the individual into a “psychological advantage” to be fully converted into action capital for career development. This also suggests to career counseling practitioners that mere supplementation of material resources (such as scholarships) may not be enough to completely bridge the class gap; it is also necessary to simultaneously focus on the subjective social perception and psychological empowerment of youth from lower-class backgrounds. Therefore, it is crucial to emphasize that this “association pathway” is a statistical construct derived from cross-sectional data. It illustrates a strong association between variables but does not, and cannot, represent a verified causal process over time, which awaits future verification from longitudinal studies.

In contrast, the Kazakhstani sample presents a completely different picture. The explanatory strength of social capital (interpersonal networks) (69.1%) replaced that of economic capital, becoming the core factor associated with career self-determination. This finding is consistent with the still-resilient family and tribal cultural traditions in Kazakhstani society. In such a social structure, social networks formed by kinship and geographical ties have a more important association with information transmission, opportunity recommendation, and even risk sharing than market-based monetary exchange. For Kazakhstani youth, “who you know” may be more directly convertible into career development certainty and their internal psychological capital than “how much money you have” (Gheyassi and Alambeigi, 2024). This also reminds us that in understanding the career ecology of Central Asian societies, we cannot simply apply the analytical frameworks from Western or East Asian market economies; we must fully consider their unique social relationship patterns.

### 5.2 The role of cultural values: a general negative main effect

Interestingly, the hypothesis of this study regarding the moderating association of traditional cultural values (H2) was not supported. Both in the combined cross-national sample and in the supplementary tests on the independent samples of China and Kazakhstan, traditional cultural values showed a significant negative main association with career self-determination, but their interaction term with family SES (using subjective class perception as the focused proxy) was not significant. This means that regardless of high or low family socioeconomic status, youth who highly endorse traditional values have lower levels of autonomy and intrinsic motivation in their career decisions. As for modern individualistic values, as this study only used a single exploratory item for measurement, its reliability limited our ability to conduct in-depth hypothesis testing and inference on its potential moderating role. This methodological limitation should be addressed in future research using more complete scales.

This result can perhaps be interpreted from two perspectives. First, traditional cultural values, as a cultural script deeply embedded in an individual's cognitive schema, have a universal, cross-class penetrating nature in defining the “individual-collective” relationship. It emphasizes that individuals should serve the overall interests of the family, placing personal choices within the framework of elder expectations and social evaluation. This disciplining force may be equally effective for youth from high-SES and low-SES families, causing them to unconsciously engage in “self-censorship” when making decisions. This is highly consistent with Hofstede's (2001) core assertion about the cross-class universal permeability of collectivist cultural values: Collectivism is not an “exclusive cultural script” of a particular class but acts as a diffuse social norm that systematically constrains the autonomous decision-making space of individuals from different socioeconomic backgrounds. Schwartz (1992) also pointed out that cultural values play more of a “background constraining variable” rather than a “contextual moderating variable” role in individual behavior, meaning they exert a continuous association under all conditions, rather than being activated only when specific resource conditions appear. This robust negative main effect, coupled with the lack of moderation, provides a highly critical nuance to classical cultural theories. While previous literature often assumes that cultural values only “trigger” under specific resource constraints, our findings align with a more structuralist view: traditional disciplinary forces are ubiquitous. They permeate cognitive schemas so deeply that even high-SES privileges cannot shield youths from collective conformity pressures, adding crucial empirical depth to the interpretations offered by Schwartz (1992). The significant negative main effect of traditional values in this study, rather than a significant interaction effect, provides empirical evidence from the China-Kazakhstan cross-cultural context for this theoretical position.

Second, the non-significant interaction effect may also indicate that the influence of family SES on career choice is so direct and “materialistic” that it is difficult to form an effective moderating lever at the more “abstract” level of cultural values. In other words, regardless of whether a young person internally agrees with tradition, when faced with real economic barriers or network opportunities, the influence of these “hard resources” may transcend differences at the conceptual level. This interpretation corresponds with Lin's (2001) argument about the priority of structural capital over cultural norms: under conditions of sufficiently strong resource constraints, the “hard boundaries” at the material level often have a stronger association with individual behavior than the “soft guidance” at the conceptual level.

Thirdly, from a methodological perspective, the lack of support for the moderation effect might also be due to the limited scope of subjective class perception as a proxy for overall SES. The moderation test for H2 in this study primarily focused on the subjective class dimension. Future research needs to introduce more dimensions of objective capital indicators (such as separating the aforementioned economic and social capital) for a more comprehensive multi-dimensional moderation effect analysis.

### 5.3 Deep socio-cultural roots of cross-national differences

The core value of this study lies in revealing the significant structural differences in the “family capital-career psychology” conversion path between China and Kazakhstan through direct cross-national data comparison. The strong support for hypotheses H3 and H4 reflects the different modernization paths and socio-cultural soils of the two countries.

The findings suggest that the overwhelming advantage of economic capital in the Chinese sample (H3) is a microcosm of its market-oriented reforms and highly competitive “involution” over the past few decades. Social realities such as the industrialization of education, high housing prices, and the urban-rural dual structure have made economic resources highly correlated with a family's ability to create career development opportunities for their children (Liu and Zang, 2024). In this structural context, the association between economic capital and children's career development opportunities has been continuously documented in existing sociology of education research (Liu and Zang, 2024). Parents' efforts to accumulate wealth show a close positive correlation with their children's access to educational and career advantages, and the children themselves are well aware of the importance of an economic foundation, thus being more sensitive to economic returns in their decision-making.

It appears that the strong association of social capital in the Kazakhstani sample (H4) reflects the unique path of a post-Soviet Central Asian country in its modernization transition. On the one hand, the market economy has not yet fully penetrated all aspects of social relations; on the other hand, tribal and clan relationship networks originating from nomadic traditions still have important social functions in contemporary Kazakhstani society (Khussainova et al., 2023). This “relational society” provides important informal social security and opportunity networks for individuals within it. For Kazakhstani youth, integrating into and maintaining their family and community relationships is key to obtaining trust, information, and career opportunities. As the data presented earlier shows, although there is no significant difference in the absolute quantity of social capital reported by Chinese and Kazakhstani youth, in Kazakhstan, these social network resources can be more efficiently associated with psychological motivation for career decisions (with a relative explanatory weight of up to 69.1%). This cross-national heterogeneity in resource “conversion efficiency” is a direct reflection of how the deep cultural context is related to the association mechanism of family capital. This is also consistent with recent local empirical findings on the extreme dependence of the country's graduates on psychological capital and social networks (Kim et al., 2024). Therefore, their career decision-making process is naturally more deeply embedded in the operating logic of this social capital.

Synthesizing the above findings, there is a structural difference in the types of family resources that youth from the two countries rely on when facing career choices: The career decisions of Chinese youth are more strongly associated with the material conditions and market access resources provided by family economic capital; the career decisions of Kazakhstani youth are more strongly associated with the information channels and opportunity recommendation mechanisms provided by family social networks. This is consistent with the differences between the two countries in their degree of marketization and social trust structures.

## 6 Conclusion, practical implications, and future prospects

### 6.1 Research conclusions

By conducting a cross-cultural survey and analysis of 1,020 valid questionnaires from youth in China and Kazakhstan, this study draws the following core conclusions:

First, there is a universal and significant positive association between family socioeconomic status and the level of career self-determination in youth. Higher family objective capital (economic capital, social capital) and a more advantageous subjective class perception are significantly related to stronger career decision-making autonomy, competence, and more active exploration behavior. Further mediation analysis shows that family objective capital can also have an indirect association with career self-determination through the psychological intermediate link of subjective social class perception, revealing an association pathway where an objective capital advantage is internalized into psychological perception, which in turn is associated with career action.

Second, traditional cultural values (emphasizing conformity to the collective and family) are significantly negatively associated with career self-determination, but their moderating association with the relationship between family SES and career self-determination is not significant in the combined sample, manifesting as a universal negative main association across social classes.

Third, and most importantly, the findings revealed a significant cross-cultural difference in the association mechanism of family capital's effect: In China, economic capital is the primary factor associated with youth career self-determination; whereas in Kazakhstan, social capital (interpersonal networks) has the strongest association. This structural difference is consistent with the differences in social structure, modernization paths, and cultural traditions between the two countries.

### 6.2 Practical implications

The findings of this study have clear practical guidance significance for career education and youth counseling work in both China and Kazakhstan:

#### 6.2.1 Implications for China

Career Education Level: Given the powerful association of economic factors, career counseling in schools and social institutions should pay special attention to students from low-economic-capital families. They should be provided with more information compensation, internship opportunities, and scholarship support to compensate for their disadvantages in resource acquisition and to prevent them from prematurely abandoning personal interests due to economic pressure.

Family Education Level: The research results indicate that subjective class perception plays an important psychological mediating role between objective capital and career self-determination. This means that while providing material support for their children, parents should also pay attention to their children's psychological perception of their own social position and development potential. For families with relatively limited economic capital, parents can help their children establish a positive class self-positioning through positive verbal reinforcement and sharing their own experiences of overcoming adversity, among other strategies, thereby buffering the negative association between insufficient objective resources and career autonomy to some extent.

#### 6.2.2 Implications for Kazakhstan

Career Education Level: The career counseling system should acknowledge and make good use of the positive association of social networks. For example, students can be encouraged to find career mentors and practical opportunities through family and community relationships. At the same time, it is also necessary to guide students to develop critical thinking, to examine the potential limitations of career paths brought by relational networks, and to encourage them to break through traditional circles and explore broader modern career fields.

Social Policy Level: Efforts should be made to build a fairer and more transparent employment market, gradually weakening the excessive association of informal social relationships in career access, and providing a more merit- and talent-based competitive platform for all youth.

### 6.3 Research limitations and future prospects

Although this study has achieved valuable findings in its comparison of the factors associated with career self-determination between Chinese and Kazakhstani students, it still has some limitations that can provide directions for future research:

Limitations of Cross-Sectional Design: It should be noted that this study used a cross-sectional survey design, and all conclusions reflect the association patterns between variables rather than causal relationships. The causal mechanisms await further verification by future longitudinal studies. Future research could adopt a longitudinal study design to examine how family capital and cultural values dynamically associated with the developmental trajectory of career self-determination over a longer time span.

Problem of Sample Representativeness: Although the sample size is large, sampling mainly relied on online channels, which may not have fully covered all regions and social strata of the two countries, especially in remote rural areas. Future research could use more representative stratified random sampling methods.

Limitations in Construct Measurement: This study faced limitations in the measurement of key theoretical constructs. Firstly, “culture” was operationalized into several core value dimensions, which to some extent simplifies its complexity. Particularly for “modern individualistic values,” only a single item was used for assessment, resulting in insufficient reliability and limiting in-depth exploration of its role. Secondly, as justified in the theoretical framework, this study deliberately focused on the more directly measurable forms of economic, social, and cultural capital to ensure a tractable cross-cultural comparison. While this approach enhanced the study's empirical clarity, it represents a necessary trade-off. Consequently, the association of symbolic capital (e.g., family prestige and honor) was not independently assessed. The omission of this dimension means that the model may not fully capture its nuanced associations, which could be a particularly relevant factor in both cultural contexts. Future research should develop or adopt rigorously validated multi-dimensional, multi-item localized scales for both cultural values and all dimensions of family capital, including symbolic capital.

Potential Risk of Common Method Bias: Although this study ruled out serious common method bias through procedural controls and statistical tests (Harman + CFA), since all data were derived from self-reports from a single source, the minor association of bias cannot be 100% excluded.

Expansion of Research Scope: There are many countries along the “Belt and Road” with diverse cultures. Future research could extend the framework of this study to more Central Asian, Southeast Asian, and even Central and Eastern European countries to build a more universal and explanatory cross-cultural model of career development.

Finally, the results of the relative weight analysis are constrained by the set of predictor variables chosen by the researcher. This study only included three types of family capital dimensions and did not control for other important potential variables such as school education quality, peer influence, and personal traits. Therefore, the relative contribution proportions of each capital dimension should not be interpreted in an absolute sense as their objective ranking among all possible associated factors.

## Data Availability

The original contributions presented in the study are included in the article/Supplementary material, further inquiries can be directed to the corresponding author.
