# Supplementary material for: The relationship between family socioeconomic status and cultural background on the career self-determination of Chinese and Kazakhstani students
Source: Front Psychol. 2026 Jun 22;17:1849182. doi: 10.3389/fpsyg.2026.1849182 (PMC13333450; doi:10.3389/fpsyg.2026.1849182)
Supplement: Supplementary file 1 [file Data_Sheet_1.zip › Supplementary_S1_Data_Codebook.docx]

### Data Codebook **Missing Values:** The dataset contains no missing values. All included cases completed the survey in its entirety. **General Scaling Note:** Except for demographic items, all psychological and capital-related constructs were measured on a 5-point Likert scale (1 = *Strongly Disagree* / *Cannot at all*, 5 = *Strongly Agree* / *Can fully*), unless otherwise specified.

### 1. Demographic Variables (Basic Information)

| Variable Name | Item No. | Variable Label / Description | Coding Scheme (Values & Definitions) |
| --- | --- | --- | --- |
| Subject_ID | N/A | Anonymized Subject Identifier | Continuous integers from 1 to 1020 |
| Q2_Country | Q2 | Nationality / Country of Origin | 1 = China; 2 = Kazakhstan |
| Q3_Gender | Q3 | Gender | 1 = Male; 2 = Female; 3 = Other |
| Q4_Age | Q4 | Age | Continuous (entered as exact numerical age, e.g., 18, 22) |
| Q5_Edu | Q5 | Current Educational Stage | 1 = High School; 2 = University (Y1-Y2); 3 = University (Y3-Y4); 4 = Vocational College; 5 = Master’s or above |
| Q6_Location | Q6 | Family Primary Residence Location | 1 = Tier-1 / Major metropolitan cities; 2 = Tier-2/3 / Medium to small cities; 3 = County / Town / Rural areas |

### 2. Family Socioeconomic Status & Objective Capital

| Variable Name | Item No. | Variable Label / Description | Coding Scheme (Values & Definitions) |
| --- | --- | --- | --- |
| Q7_FaEdu | Q7 | Father’s Highest Education Level | 1 = Primary school or below; 2 = Junior high; 3 = High school / Voc. school; 4 = Associate degree; 5 = Bachelor’s degree; 6 = Master’s degree or above |
| Q8_MoEdu | Q8 | Mother’s Highest Education Level | 1 = Primary school or below; 2 = Junior high; 3 = High school / Voc. school; 4 = Associate degree; 5 = Bachelor’s degree; 6 = Master’s degree or above |
| Q9_Income | Q9 | Average Monthly Family Income | 1 = Lowest income bracket; 2 = Lower-middle; 3 = Upper-middle; 4 = Highest income bracket (equivalized for respective national currencies) |
| Q10_SubSES | Q10 | Subjective Social Status (Ladder) | 1 to 10 (1 = Lowest societal tier, 10 = Highest/top societal tier) |
| Q11_EconSup | Q11 | Economic Capital Support | 1 (*Cannot at all*) to 5 (*Can fully provide funds/allowance*) |
| Q12_InfoSup | Q12 | Informational Capital Support | 1 (*Cannot at all*) to 5 (*Can fully provide career guidance/advice*) |
| Q13_SocSup | Q13 | Social Network Capital Support | 1 (*Cannot at all*) to 5 (*Can fully provide direct career opportunities/networking*) |

### 3. Psychological Constructs & Cultural Values

*(Note: All items in this section are coded using a 5-point Likert scale: 1 = Strongly Disagree, 2 = Disagree, 3 = Neutral, 4 = Agree, 5 = Strongly Agree)*

| Variable Name | Item No. | Sub-dimension assigned | Item Description / Measuring Concept |
| --- | --- | --- | --- |
| Q14_Trad1 | Q14 | Cultural Values (Collectivism/Family) | Prioritizing family interests over personal career interests. |
| Q15_Trad2 | Q15 | Cultural Values (Elder Obedience) | Respecting elders’ career advice or arrangements. |
| Q16_Trad3 | Q16 | Cultural Values (Face/Prestige) | Unstable careers affect the family’s prestige (‘Face’). |
| Q17_Trad4 | Q17 | Cultural Values (Social Status) | Choosing a “respectable” career over a preferred one. |
| Q18_Mod1 | Q18 | Cultural Values (Individualism/Modern) | Defining success individually, disregarding traditional standards. *(Note: Often used as a reverse or contrasting metric to Q14-Q17)* |
| Q19_Auto1 | Q19 | Career Decision Autonomy | Career goals are self-initiated, not imposed by others. |
| Q20_Auto2 | Q20 | Career Decision Autonomy | Freedom to express career thoughts without pressure from others’ expectations. |
| Q21_Auto3 | Q21 | Career Decision Autonomy | Courage to persist with a career choice despite family opposition. |
| Q22_Auto4 | Q22 | Career Decision Autonomy | Valuing internal passion over high salary and status. |
| Q23_Auto5 | Q23 | Career Decision Autonomy | Ideal career must actualize personal talents and inner values. |
| Q24_Expl1 | Q24 | Career Exploration Behavior | Proactively participating in courses/reading to understand real work environments. |
| Q25_Expl2 | Q25 | Career Exploration Behavior | Actively consulting professionals/seniors for career planning advice. |
| Q26_Expl3 | Q26 | Career Exploration Behavior | Actively seeking out internships, career fairs, or experiential activities. |
| Q27_Effi1 | Q27 | Career Decision Self-Efficacy | Clear awareness of strengths/weaknesses and accurately judging suitable industries. |
| Q28_Effi2 | Q28 | Career Decision Self-Efficacy | Ability to make firm, unregrettable decisions among risky/tempting options. |
| Q29_Effi3 | Q29 | Career Decision Self-Efficacy | High confidence in overcoming future competitive or uncertain career challenges. |
