# Supplementary material for: The relationship between family socioeconomic status and cultural background on the career self-determination of Chinese and Kazakhstani students
Source: Front Psychol. 2026 Jun 22;17:1849182. doi: 10.3389/fpsyg.2026.1849182 (PMC13333450; doi:10.3389/fpsyg.2026.1849182)
Supplement: Supplementary file 1 [file Data_Sheet_1.zip › Supplementary_Material_S2_Questionnaires_Bilingual.docx]

**Supplementary Material S2: Survey Questionnaires used in the Study**

*Translation Procedure Note: To ensure semantic and conceptual equivalence across different cultural contexts, the original questionnaire was developed in Chinese. It was then adapted into English as an intermediary standard, and subsequently translated into Russian for the Kazakhstani participants. A strict translation and back-translation procedure was conducted by bilingual academic professionals in psychology and sociology from both China and Kazakhstan. No major discrepancies were found, ensuring measurement equivalence.*

**Preface and Informed Consent Form**

Hello! Our research team is conducting a cross-cultural academic study on how family socioeconomic status and cultural background influence the career choices of young students. The questionnaire is completely anonymous. All data will be used solely for overall statistical analysis and academic publication and will be kept strictly confidential. There are no right or wrong answers; your genuine thoughts are extremely important to this study. Your participation is entirely voluntary, and you may withdraw at any time during the process. The survey is expected to take about 5 minutes to complete. Thank you for your support!

**Part I: Informed Consent**

1. Have you read and understood the above instructions, and do you voluntarily agree to participate in this survey? [Single-choice question]
   □ I agree to participate and begin the survey
   □ I do not agree (selecting this will end the survey immediately) (Note: The system is set to redirect to the closing message)

**Part II: Basic Information**

1. Which country are you in? [Single-choice question]
   □ China
   □ Kazakhstan
2. What is your gender? [Single-choice question]
   □ Male
   □ Female
   □ Other (please specify: ______)
3. What is your age? [Fill-in-the-blank question]
   ______ years old
4. What is your current educational stage? [Single-choice question]
   □ High school
   □ First or second year of university (exploration period)
   □ Third or fourth year of university (decision period)
   □ Junior college / Vocational school
   □ Master’s degree or above
5. What type of area does your family permanently reside in? [Single-choice question]
   □ Tier-1 city / major metropolitan area (e.g., Beijing, Shanghai, Guangzhou, Shenzhen in China; Astana, Almaty in Kazakhstan)
   □ Tier-2/3 city / small to medium-sized city
   □ County / township / rural area

**Part III: Family Socioeconomic Status (SES)**
(This data is crucial. Please choose according to your family’s actual situation. Your information will be kept confidential.)

1. What is your father’s highest level of education? [Single-choice question]
   □ Primary school or below □ Junior high school □ High school / Vocational high school □ Junior college □ Bachelor’s degree □ Master’s degree or above
2. What is your mother’s highest level of education? [Single-choice question]
   □ Primary school or below □ Junior high school □ High school / Vocational high school □ Junior college □ Bachelor’s degree □ Master’s degree or above
3. What is your family’s average monthly total income? (Please choose according to the currency of your country) [Single-choice question]
   (Options for Chinese respondents)
   □ 5,000 RMB or less
   □ 5,001 - 15,000 RMB
   □ 15,001 - 30,000 RMB
   □ 30,001 RMB or more

(Options for Kazakhstani respondents)
□ 150,000 Tenge or less
□ 150,000 - 300,000 Tenge
□ 300,000 - 600,000 Tenge
□ 600,000 Tenge or more

1. [Subjective Social Ladder] Imagine society is a ladder with 10 rungs. Rung 10 represents people with the highest social status, the most wealth, and the highest education. Rung 1 represents people with the lowest social status, the least wealth, and the lowest education. Where do you think your family currently stands on this ladder? [Single-choice question, 1-10 scale]
   □ 1 (Lowest rung) □ 2 □ 3 □ 4 □ 5 □ 6 □ 7 □ 8 □ 9 □ 10 (Highest rung)

(Based on the actual situation, please rate the following dimensions of objective family support. 1=Not at all able, 5=Completely able) [Matrix scale questions]

1. Can your family provide financial support for your career development (e.g., cover costs for studying abroad, living expenses during an unpaid internship, or seed funding for a startup)? [Single-choice option: 1 to 5]
2. Can your parents provide you with professional career information or guidance (e.g., clearly analyze industry prospects, guide resume writing or interviews)? [Single-choice option: 1 to 5]
3. Can your parents directly provide you with career opportunities through their social connections (e.g., recommend high-quality internships, provide internal job referrals)? [Single-choice option: 1 to 5]

**Part IV: Cultural Value Identification**
(There are no right or wrong answers to the following statements. Please choose based on your true level of agreement: 1=Strongly disagree, 5=Strongly agree) [Matrix scale questions]

1. In my career choice, I should prioritize the overall interests of my family (e.g., taking care of parents, stable income) rather than solely pursuing personal interests.
2. Respecting elders’ advice or even arrangements regarding one’s career is a very important cultural tradition.
3. If a young person chooses an unstable or low-status job, it will affect the entire family’s reputation (or “face”) among relatives and friends.
4. I believe choosing a “decent” job that is widely respected in my country’s society is more important than choosing something I simply like.
5. I believe the standards of career success should absolutely be defined by myself, rather than following the uniform traditional cognition of society.

**Part V: Career Self-Determination (Reality Perception and Behavior)**
(This section measures your actual decision-making state. Based on your actual situation, please choose the frequency or level of agreement that best fits you: 1=Strongly disagree/Never, 5=Strongly agree/Always) [Matrix scale questions]

[A.Career Decision-Making Autonomy]

1. My career development goals are set by myself proactively, not by following the crowd or being imposed by others.
2. When choosing my future career path, I can freely express my own ideas without feeling pressured by others’ expectations.
3. Even if my family strongly opposes the career field I want to pursue, I still have the courage to and will insist on my choice.
4. Compared to salary and status, I value whether a job can make me feel passionate about my work every day when choosing a career.
5. My ideal career must be a platform that allows me to fully utilize my talents and realize my inner self-worth.
6. Career Exploration Behavior]
7. I proactively read books, browse industry websites, or attend courses to gain a deeper understanding of the real work status of different professions.
8. I have proactively sought out professional teachers, experienced practitioners, or senior students to have in-depth discussions about career choices and planning.
9. I have proactively sought and participated in career experiences, company internships, or related practical activities (such as campus recruitment fairs, industry seminars).
10. Career Decision-Making Competence]
11. After evaluation, I am very clear about my core strengths and weaknesses and can accurately judge which industries are truly suitable for me.
12. When faced with multiple tempting or risky career options, I am capable of making a clear decision that I will not regret.
13. I am confident in my ability to cope with the difficulties I will inevitably encounter in future career choices (such as fierce competition, rejection, environmental uncertainty).

[End of Questionnaire]
You have completed the survey! Thank you again for taking your valuable time to participate in this research. Your feedback will be of great help to cross-cultural youth career psychology research. We wish you success in your studies and a bright future!

### **Введение и информированное согласие**

### Здравствуйте! Наша исследовательская группа проводит межкультурное академическое исследование о том, как социально-экономическое положение семьи и культурный фон влияют на профессиональный выбор молодежи. Опрос проводится полностью анонимно. Все данные будут использованы исключительно для общего статистического анализа и академических публикаций и будут храниться в строгой конфиденциальности. Здесь нет правильных или неправильных ответов; ваше искреннее мнение чрезвычайно важно для данного исследования. Ваше участие является полностью добровольным, и вы можете прервать заполнение анкеты в любой момент. Опрос займет около 5 минут. Спасибо за вашу поддержку!

### **Часть I: Информированное согласие**

### Прочитали ли вы и поняли ли вышеуказанные инструкции и добровольно ли соглашаетесь участвовать в этом опросе? [Один вариант ответа] □ Согласен(-на) участвовать, начать опрос □ Не согласен(-на) (выбор этого варианта немедленно завершит опрос) (Примечание: система настроена на переход к заключительному сообщению)

### **Часть II: Общая информация**

1. В какой стране вы находитесь? [Один вариант ответа]
   □ Китай
   □ Казахстан
2. Ваш пол? [Один вариант ответа]
   □ Мужской
   □ Женский
   □ Другой (укажите: ______)
3. Ваш возраст? [Вопрос с полем для ввода]
   ______ лет
4. Ваш текущий уровень образования? [Один вариант ответа]
   □ Средняя школа
   □ Первый или второй курс университета (период поиска)
   □ Третий или четвертый курс университета (период принятия решений)
   □ Колледж / профессионально-техническое училище
   □ Магистратура или выше
5. Тип местности, где постоянно проживает ваша семья? [Один вариант ответа]
   □ Город первого уровня / крупный мегаполис (например, Пекин, Шанхай, Гуанчжоу, Шэньчжэнь в Китае; Астана, Алматы в Казахстане)
   □ Город второго/третьего уровня / малый или средний город
   □ Уездный город / поселок / сельская местность

**Часть III: Социально-экономическое положение семьи (СЭП)**
(Эти данные чрезвычайно важны. Пожалуйста, выберите вариант, соответствующий реальному положению вашей семьи. Вся информация строго конфиденциальна.)

1. Самый высокий уровень образования вашего отца? [Один вариант ответа]
   □ Начальная школа или ниже □ Неполная средняя школа □ Средняя школа / среднее специальное □ Колледж (специалитет) □ Бакалавриат □ Магистратура или выше
2. Самый высокий уровень образования вашей матери? [Один вариант ответа]
   □ Начальная школа или ниже □ Неполная средняя школа □ Средняя школа / среднее специальное □ Колледж (специалитет) □ Бакалавриат □ Магистратура или выше
3. Примерный среднемесячный общий доход вашей семьи? (Пожалуйста, выберите в соответствии с валютой вашей страны) [Один вариант ответа]
   (Варианты для респондентов из Китая)
   □ 5 000 юаней или меньше
   □ 5 001 - 15 000 юаней
   □ 15 001 - 30 000 юаней
   □ 30 001 юаней или больше

(Варианты для респондентов из Казахстана)
□ 150 000 тенге или меньше
□ 150 000 - 300 000 тенге
□ 300 000 - 600 000 тенге
□ 600 000 тенге или больше

1. [Субъективная социальная лестница] Представьте, что общество — это лестница с 10 ступенями. 10-я ступень представляет людей с самым высоким социальным статусом, наибольшим богатством и самым высоким уровнем образования. 1-я ступень представляет людей с самым низким социальным статусом, наименьшим богатством и самым низким уровнем образования. Как вы думаете, на какой ступени этой лестницы находится ваша семья в настоящее время? [Один вариант ответа, шкала от 1 до 10]
   □ 1 (самая нижняя ступень) □ 2 □ 3 □ 4 □ 5 □ 6 □ 7 □ 8 □ 9 □ 10 (самая верхняя ступень)

(Исходя из реальной ситуации, пожалуйста, оцените следующие аспекты объективной поддержки со стороны семьи. 1=Совершенно не может, 5=Полностью может) [Матричные вопросы]

1. Может ли ваша семья оказать финансовую поддержку для вашего карьерного развития (например, покрыть расходы на обучение за границей, расходы на проживание во время неоплачиваемой стажировки или предоставить стартовый капитал для бизнеса)? [Один вариант ответа: от 1 до 5]
2. Могут ли ваши родители предоставить вам профессиональную информацию или консультацию по карьере (например, четко проанализировать перспективы отрасли, помочь с составлением резюме или подготовкой к собеседованию)? [Один вариант ответа: от 1 до 5]
3. Могут ли ваши родители напрямую предоставить вам карьерные возможности через свои социальные связи (например, порекомендовать качественную стажировку, предоставить внутреннюю рекомендацию на работу)? [Один вариант ответа: от 1 до 5]

**Часть IV: Определение культурных ценностей**
(В следующих утверждениях нет правильных или неправильных ответов. Пожалуйста, выберите степень вашего согласия: 1=Категорически не согласен(-на), 5=Полностью согласен(-на)) [Матричные вопросы]

1. При выборе профессии я должен(-на) в первую очередь учитывать общие интересы семьи (например, забота о родителях, стабильный доход), а не просто следовать личным интересам.
2. Уважение к советам старших по поводу профессии или даже к их выбору за меня является очень важной культурной традицией.
3. Если выбранная молодым человеком профессия нестабильна или имеет низкий социальный престиж, это повлияет на репутацию (или «лицо») всей семьи среди родственников и друзей.
4. Я считаю, что выбрать «достойную» профессию, уважаемую в обществе моей страны, важнее, чем выбрать то, что мне просто нравится.
5. Я считаю, что критерии карьерного успеха должны определяться исключительно мной, а не следовать общепринятым традиционным представлениям общества.

**Часть V: Карьерное самоопределение (Восприятие реальности и поведение)**
(Этот раздел измеряет ваше реальное состояние при принятии решений. Исходя из реальной ситуации, выберите наиболее подходящую для вас частоту или степень согласия: 1=Категорически не согласен(-на)/Никогда, 5=Полностью согласен(-на)/Всегда) [Матричные вопросы]

[А. Автономия в принятии карьерных решений]

1. Мои цели в карьерном развитии я ставлю перед собой сам(-а) proactively, а не следую за толпой или под давлением других.
2. При выборе будущего карьерного пути я могу свободно выражать свои мысли, не чувствуя давления из-за ожиданий других.
3. Даже если моя семья категорически против той сферы деятельности, которой я хочу заниматься, у меня все равно хватит смелости настоять на своем выборе.
4. При выборе профессии для меня важнее, чтобы работа вызывала у меня ежедневный энтузиазм, чем зарплата и статус.
5. Моя идеальная профессия должна быть платформой, которая позволит мне полностью раскрыть свои таланты и реализовать свою внутреннюю ценность.

[Б. Поведение в области карьерного поиска]
24. Я активно читаю книги, просматриваю отраслевые сайты или посещаю курсы, чтобы глубже понять реальное состояние дел в различных профессиях.
25. Я активно обращался(-ась) к профессиональным преподавателям, опытным специалистам или старшим студентам для глубокого обсуждения вопросов выбора и планирования карьеры.
26. Я активно искал(-а) и участвовал(-а) в профессиональных стажировках, практиках в компаниях или связанных с этим мероприятиях (таких как ярмарки вакансий, отраслевые семинары).

[В. Компетентность в принятии карьерных решений]
27. После самоанализа я очень хорошо понимаю свои сильные и слабые стороны и могу точно определить, какие отрасли мне действительно подходят.
28. Столкнувшись с несколькими соблазнительными или рискованными вариантами карьеры, я способен(-на) принять четкое решение, о котором не буду жалеть.
29. Я уверен(-а) в своей способности справиться с трудностями, с которыми я неизбежно столкнусь при выборе будущей карьеры (такими как жесткая конкуренция, отказы, неопределенность).

[Завершение опроса]
Опрос завершен! Еще раз искренне благодарим вас за то, что вы уделили свое драгоценное время участию в этом исследовании. Ваши ответы окажут огромную помощь в межкультурном исследовании психологии карьеры молодежи. Желаем вам успехов в учебе и светлого будущего

**卷首语与知情同意书**
您好！本研究团队正在进行一项关于家庭社会经济地位与文化背景如何影响青年学生职业选择的跨文化学术研究。问卷采用完全匿名方式收集，所有数据仅用于总体统计分析和学术发表，严格保密。答案无对错之分，您的真实想法对本研究极其重要。您可完全自愿参与，并可在填写过程中随时退出。填写预计需要 5 分钟。感谢您的支持！

**第一部分：知情同意**

1.您是否已阅读并理解上述说明，完全自愿同意参与本次调查？【单选题】
□ 同意参与，开始答题
□ 不同意（选择此项将直接结束问卷） *(注：问卷星后台设置跳转到结束语)*

**第二部分：基本信息**
2. 您所在的国家是？【单选题】
□ 中国
□ 哈萨克斯坦

3您的性别是？【单选题】
□ 男
□ 女
□ 其他（请注明：______）

4.您的年龄是？【填空题】
______岁

5.您目前的教育阶段属于？【单选题】
□ 高中阶段
□ 大学一、二年级（探索期）
□ 大学三、四年级（决策期）
□ 大专 / 高职学校
□ 硕士研究生及以上

6.您家庭常住的地区类型是？【单选题】
□ 一线城市 / 主要大城市（如中国的北上广深、省会；哈萨克斯坦的阿斯塔纳、阿拉木图）
□ 二三线城市 / 中小城市
□ 县城 / 乡镇 / 农村

**第三部分：家庭社会经济地位 (SES)**
*(此项数据极为关键，请根据家庭实际情况放心选择)*

7.您父亲的最高学历是？【单选题】
□ 小学及以下 □ 初中 □ 高中 / 中专 □ 专科 □ 本科 □ 硕士及以上

8.您母亲的最高学历是？【单选题】
□ 小学及以下 □ 初中 □ 高中 / 中专 □ 专科 □ 本科 □ 硕士及以上

9.您家庭的月均总收入约为？（请按您所在国家的货币情况选择）【单选题】
*(中国受访者选项)*
□ 5,000 元人民币及以下
□ 5,001 - 15,000 元人民币
□ 15,001 - 30,000 元人民币
□ 30,001 元人民币及以上
*(哈萨克斯坦受访者选项)*
□ 15 万坚戈及以下
□ 15 万 - 30 万坚戈
□ 30 万 - 60 万坚戈
□ 60 万坚戈及以上

10.**【主观社会阶层定位】** 想象社会是一个包含 1-10 层的梯子，第10层代表社会地位最高、最富有、受教育程度最高的人；第1层代表社会地位最低、最贫穷、受教育程度最低的人。请问您认为您的家庭目前处在这个梯子的哪一层？【单选题，1-10分】
□ 1（最底层） □ 2 □ 3 □ 4 □ 5 □ 6 □ 7 □ 8 □ 9 □ 10（最顶层）

*(请根据实际情况，对以下家庭客观支持维度进行评分，1=完全不能，5=完全能)*【矩阵量表题】
11. 您的家庭能否为您的职业发展提供经济支持（如提供出国升学费用、承担无薪实习期的生活费、创业启动金等）？【单选项：1至5分】
12. 您的父母能否为您提供专业的职业信息或指导意见（如清晰分析行业前景、指导简历或面试）？【单选项：1至5分】
13. 您的父母能否通过他们的社会人脉为您直接提供职业机会（如推荐优质实习、内部工作推荐）？【单选项：1至5分】

**第四部分：文化价值观认同**
*(以下陈述没有对错，请根据您内心的真实认同程度选择：1=完全不认同，5=完全认同)*【矩阵量表题】

14.在职业选择中，我应优先考虑家庭的整体利益（如照顾父母、稳定收入），而非仅仅追求个人兴趣。

15.尊重长辈对职业的建议甚至是安排，是一项非常重要的文化传统。

16.年轻人选择的职业如果不稳定或社会评价低，会影响整个家庭在亲友中的声望（或“面子”）。

17.我认为选择一份在本国社会被广泛尊重的“体面”职业，比选择自己单纯喜欢的更重要。

18.我认为职业成功的标准绝对应该由自己定义，而非遵循社会传统的统一认知。

**第五部分：职业自我决定（现实认知与行为）**
*(本部分测量您的现实决策状态。请根据实际情况，选择最符合您的频率或认同度：1=完全不认同/从未，5=完全认同/总是)*【矩阵量表题】

**【A. 职业决策自主性】**
19. 我的职业发展目标是我自己主动设定的，而不是随大流或他人强加给我的。
20. 在选择未来职业方向时，我能自由表达自己的想法，不会因为承担着他人的期望而感到压力。
21. 即使家人强烈反对我想从事的职业领域，我也依然有勇气并会坚持自己的选择。

22. 相比于薪水和地位，我选择某项职业更看重它是否能够每天让我感到内心充满工作热情。
23. 我理想中的职业，必须是一个能够让我完全发挥自身天赋、实现自我内在价值的平台。

**【B. 职业探索行为】**
24. 我会主动去查阅书籍、浏览行业网站或参加课程，以深入了解不同职业的真实工作状态。
25. 我曾主动找过专业的老师、资深从业者或学长学姐，深度探讨过职业选择与规划的问题。
26. 我主动去争取并参加过职业体验、企业实习或相关的实践活动（如校园招聘会、行业讲座）。

**【C. 职业决策胜任感】**
27. 经过评估，我非常清楚自己的核心优势和劣势，并能准确判断哪些行业真正适合自己。
28. 面对多种极具诱惑力或同时存在风险的职业选择时，我有能力做出不让自己后悔的明确决策。
29. 我对自己应对未来职业选择中必将遇到的困难（如激烈竞争、被拒、环境不确定性）充满信心。
**【问卷结束语】**
答题完毕！再次衷心感谢您抽出宝贵时间参与本项研究。您的反馈将为跨文化青年职业心理研究提供极大的帮助。祝您学业顺利，未来前程似锦！
